# Supplementary material for: Deep learning driven biosynthetic pathways navigation for natural products with BioNavi-NP
Source: Nat Commun. 2022 Jun 10;13:3342. doi: 10.1038/s41467-022-30970-9 (PMC9187661; doi:10.1038/s41467-022-30970-9)
Supplement: Supplementary file 1 — Supplementary Information [file 41467_2022_30970_MOESM1_ESM.pdf]

Deep learning driven biosynthetic pathways navigation for natural products with BioNavi-NP

Zheng *et al.*

Supplementary Methods 1. Models construction of BioNavi-NP

Model overview

Our BioNavi-NP model consists of two main components, a multi-head Transformer neural network[1] for single-step bio-retrosynthetic prediction and a Retro* searching algorithm[2] for multi-step planning (see Figure 1b). In the following sections, we will introduce the basic mechanism behind these two modules, please see the supplementary ref. 2 and ref. 3 for more details about retrosynthetic Transformer and Retro*.

Single-step implementation

For single-step retrosynthetic prediction, we adapted the Transformer architecture (Supplementary Figure 2a) to map the sequence of products to the sequence of the reactants. The structure of the Transformer network[1] follows the encoder-decoder paradigm and is trained in an end-to-end fashion. The encoder layers are input with the source molecular SMILES sequence and transform it into a latent representation. The decoder combines the latent output from the encoder and the decoder's preceding output to predict the target molecular SMILES sequence. The training goal is to minimize the gap between predicted molecular SMILES sequence and target molecular SMILES sequence so that the model can finally infer accurate precursors.

For a particular source sequence, the training objective is to minimize the cross-entropy loss function:

$$\mathcal{L}(y,m) = -\sum_{i=1}^{K} y_i \log(m_i) \qquad (1)$$

where $\mathcal{L}$ denotes the cross-entropy loss, y_i is the predicted SMILES character and m_i is the target SMILES character.

In subsequent sections, we simplified this transformer-based single-step retrosynthesis model as $B(\cdot)$,

$$B(\cdot):\ t \rightarrow \{R_i, S_i, c(R_i)\}_{i=1}^{k} \qquad (2)$$

where t is a target molecule, R_i indicates reaction, S_i is a set of reactants and $c(R_i)$ is the cost of reaction R_i. k is the number of reactions output by $B(\cdot)$.

Our best-performance single-step models were trained for 48 hours on four GPU (Nvidia 2080TI) on the training set, saving one checkpoint every 10, 000 steps and averaging the last five checkpoints. Hyperparameters are selected based on the performance of the model on the validation set. A beam search procedure[4] was then used to infer multiple precursors candidates on the test set. We used the best performance model to infer the candidate sequences of precursors with a beam width of 10. As a result, the top ten candidate sequences ranked by total probability were retained.

Multi-step implementation

For multi-step planning, we adopt Retro*[2] (Supplementary Figure 2b) as the search engine to finds high-quality synthetic routes efficiently. Retro* is a best-first search algorithm, which exploits neural priors to directly optimize for the quality of the solution. It translates the search as an AND-OR tree, and learns a neural search bias with off policy data. Specifically, the search tree T is an AND-OR tree, with molecule node as 'OR' node and reaction node as 'AND' node. It starts the search tree T with a single root compound node, which is the target compound *t*. At each iteration, it selects a node u in the frontier of T (denoted as $\mathcal{F}(T)$) according to the value function. Then it expands u with the one-step model $B(u)$ and grows T with one AND-OR stump. Finally, the nodes with potential dependency on u will be updated.

Below we will provide a brief introduction of the algorithm by explaining these steps one by one:

Selection: Given a biosynthesis search tree *T*, we define the molecule nodes as $V^m(T)$ and reaction nodes as $V^r(T)$, and the total nodes in *T* will be $V(T) = V^m(T) \cup V^r(T)$. The frontier $\mathcal{F}(T) \subseteq V^m(T)$ includes all the molecular nodes in *T* that have not been expanded before. Since we want to minimize the total cost of the final solution, the next logical choice to expand is the molecular node belonging to the optimal synthesis plan.

Suppose we already have an eligible value function oracle $O_t(m|T)$ which tells us that under the current search tree *T*, the cost of the best plan that contains m for synthesizing target *t*. We can apply it to select the next node by:

$$m_{next} = \operatorname{argmin}_{m \in \mathcal{F}(T)} O_t(m|T) \qquad \textbf{(3)}$$

A proper design of such $O_t(m|T)$ is the key to achieve expected performance, which would not only improve search efficiency, but can also bring theoretical guarantees. Here we follow the original Retro* strategy which used a fully connected neural network scoring function O_m to compute the $O_t(\cdot\,|T)$ for expansion node selection with offline learning. The key idea is to train the neural network based on known reaction set and learn to maintain the partial order relationship of target molecule m_i between the best one-step solution and other solutions. The calculation is based on molecular Morgan fingerprint of target molecule m_i.

Expansion: After selecting the node m with minimum cost estimation $O_t(m|T)$, we will expand the search tree with k one-step retrosynthesis proposals from $B(m)$. In particular, for each predicted retrosynthesis reaction from $B(m)$, we initial a reaction node $R = R_i$ under node m, and for each molecule $m' \in S_i$, we create a molecule node under the reaction node R. This will generate an AND-OR stump under node m. Unlike in Monte Carlo tree search (MCTS) where multiple calls to $B(\cdot)$ is needed until a terminal state during rollout, here the expansion only requires a single call to the one-step model.

Update: Denote the biosynthesis search tree T after expansion of node m to be T'. Such expansion accumulates the corresponding cost information for single-step retrosynthesis. We apply this more direct information to update $O_t(\cdot\,|T')$ of all other related nodes to provide a more precise estimation of total cost.

Each time a full pathway is found during the tree expansion, the pathway is returned, and an additional bonus of 10 is received by the node, to allow for biasing toward similar successful pathways. At the end of the search, the most visited pathway is returned ("best"), and all pathways are returned ranked in order of decreasing $O_t(\cdot\,|T)$.

The pseudo-code of Retro* has been shown in Algorithm **1**.

Algorithm 1: $\texttt{Retro}^*(t)$

Initialize $T = (\mathcal{V}, \mathcal{E})$ with $\mathcal{V} \leftarrow \{t\}$, $\mathcal{E} \leftarrow \emptyset$;
while *route not found* **do**
  $m_{next} \leftarrow \text{argmin}_{m \in \mathcal{F}(T)} O_t(m)$;
  $\{R_i, \mathcal{S}_i, c(R_i)\}_{i=1}^{k} \leftarrow B(m_{next})$;
  for $i \leftarrow 1$ **to** k **do**
    Add R_i to T under m_{next};
    for $j \leftarrow 1$ **to** $|\mathcal{S}_i|$ **do**
      Add $\mathcal{S}_{ij}$ to T under R_i;
  Update $O_t(m)$ for m in $\mathcal{F}(T)$;
return route;

The ensemble Transformer with NP-like data augmentation shows the best performance in single-step evaluation. Therefore, we adopt the trained model as the backend of Retro*. When performing the internal multi-step evaluation, we set the beam size of Transformer, max route, iteration, max depth as 50, 100 and 10, respectively. It is worth noting that increasing the width, depth of the search and the number of iterations will certainly improve the accuracy, but the time consumption will also increase exponentially.

Training parameters and role of parameters

We utilized OpenNMT package to train the single-step Transformer neural networks with the following hyperparameters:

```
train -data data/${dataset}/${dataset} \
-save_model experiments/checkpoints/${dataset}/${dataset}_model \
-train_steps 100000 -param_init 0 -param_init_glorot -max_generator_batches 32 \
-batch_size 4096 -batch_type tokens -normalization tokens
-optim adam -adam_beta1 0.9 -adam_beta2 0.998 -decay_method noam -warmup_steps 8000\
-learning_rate 2 -label_smoothing 0.0 -report_every 1000 \
-layers 4 -rnn_size 256 -word_vec_size 256 -encoder_type transformer
-decoder_type transformer -dropout 0.1 -position_encoding -share_embeddings \
-global_attention general -global_attention_function softmax -self_attn_type scaled-dot \
-heads 8 -transformer_ff 2048
```

Role of parameters in Retro* and webserver.

a) Pathway_top_k: Number of pathway output. The ranking is based on the sum of all steps scores along the pathway.
b) Expansion_iters: Maximum number of iterations allowed for running the Tree search.
c) Expansion_top_k: Number of children a node is allowed to have. Specifically, it refers to how many precursors the transformer needs to predict in each step, and we generally choose 10 or 15.
d) Expansion_time(s): Time budget. Default run until the end, if you increase the iteration can be appropriate to increase, generally not more than 1800s
e) Max_depth: Maximum depth of the Tree (also the maximum number of pathway steps), we usually set to 10.

Supplementary Table 1. An overview on popular tools for retrobiosynthesis.

Name	Method	Planning strategy	Data source	Pathway ranking	Stereo-chemistry	Availability	Description
MRE[5]	Reaction search	Enumeration	KEGG	Host-dependent score	√	Webservice	Guiding the design and optimization of **heterologous biosynthesis** pathways
RouteSearch[6]		Branch-and-Bound	MetaCyc	Reaction costs<br>Atom mapping cost	√	Software	Computing the optimal metabolic routes in **genome-scale** reaction networks
PathPred[7]	Rule-based	Enumeration	KEGG	Structure similarity	√	Webservice	Predicting enzyme-catalyzed metabolic pathway based on **RDM patterns**
Novostoic[8]		Enumeration	MetRxn<br>KEGG<br>...(multiple)	Gibbs free energy	√	Code	Designing bioconversion routes while considering **mass conservation, cofactor balance**
Envipath[9]		Enumeration	EAWAG-BBD	Aerobic likelihood	-	Webservice	Predicting the microbial biotransformation of **organic environmental contaminants**
BNICE.ch[10] (ATLASx[11])		Enumeration	KEGG<br>MetaCyc<br>…(miltiple)	Gibbs free energy<br>Pathway length	-	Webservice	Allowing the *de novo* synthesis of metabolic pathways with **thermodynamic properties**
RetroPathRL[12]		MCTS	MetaNetX	Structure similarity<br>Sequence diversity	Option	Code	Exploring the bioretrosynthesis space using **reinforcement learning for metabolic engineering**
Bionavi-np	Rule-free	Retro*	KEGG<br>MetaCyc<br>…(miltiple)	Reaction cost<br>Host-dependent score<br>…(multiple)	√	Code<br>Webservice	Navigating retrobiosynthesis pathways for **natural products with no need of reaction rules**

Supplementary Table 2. The distribution of internal test set in different kingdoms and categories.

	Plant	Bacteria	Fungi	Animal	Others	Total
**AA/MA**	13	24	0	0	0	37
**AAs**	13	42	25	6	8	94
**CA/SA**	73	3	0	13	2	91
**MVA/MEP**	102	19	11	9	0	141
**Others**	4	6	3	0	2	15
**Total**	205	94	39	28	12	378[a]

[a] Some NPs are distributed in multiple kingdoms, and they were counted repeatedly, resulting in inconsistencies between the number of cases in the manuscript (368) and the total number here (378).

Supplementary Table 3. Performance comparisons of RetroPath 2.0, RetroPathRL and BioNavi-NP.

	LASER dataset (152)		Golden dataset (20)	
	**Success rate(%)**	**Time (h)**	**Hit rate of pathway(%)**	**Time (h)**
RetroPath 2.0	77.6	-	55	-
RetroPathRL	83.6	105	75	8
BioNavi-NP	94.7	12	65	1.5

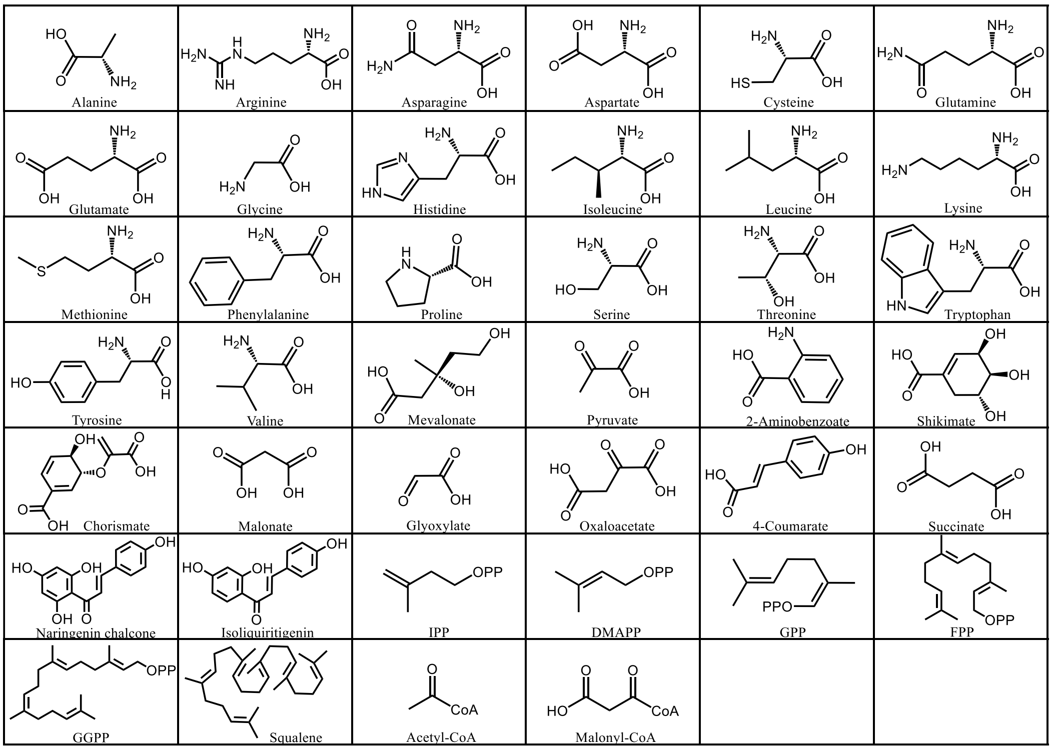

Supplementary Figure 1. The building blocks of core library used as the end point of the pathway search.

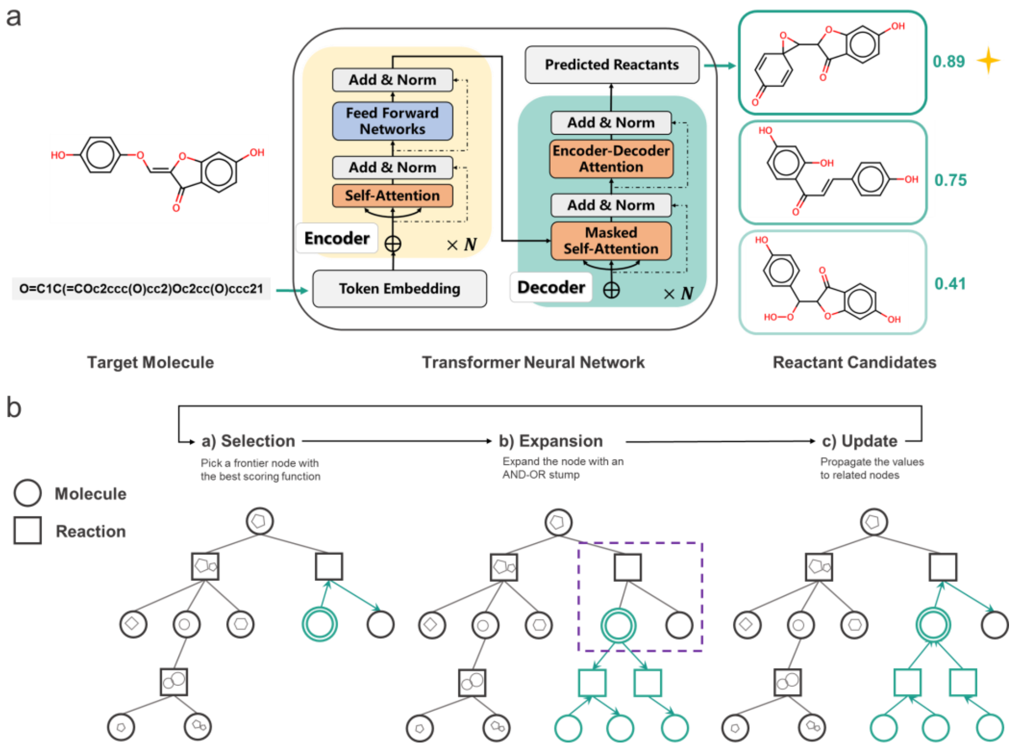

Supplementary Figure 2. A detailed illustration of Bio-Navi-NP. a Transformer model for single-step prediction. **b** Biosynthesis planning with Retro*.

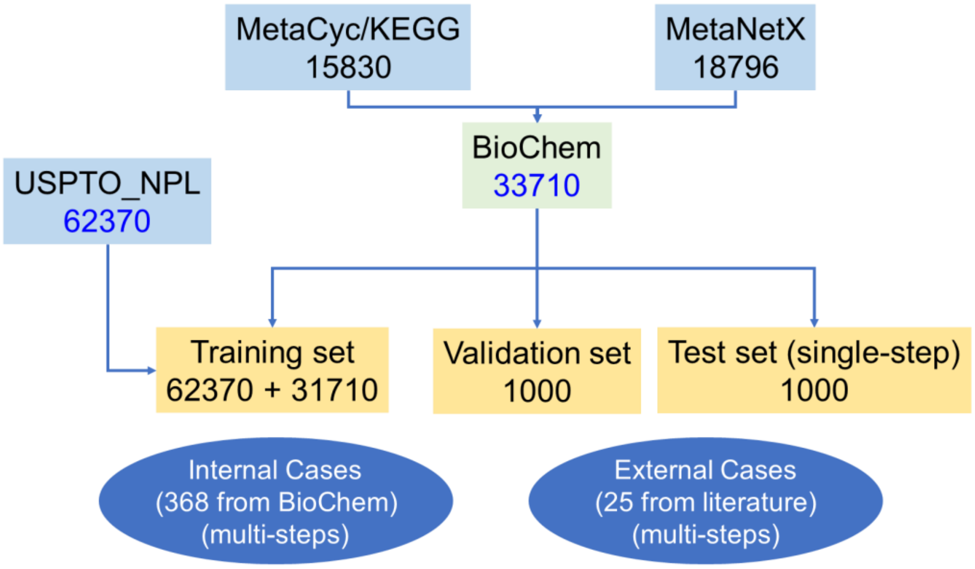

Supplementary Figure 3. Data set used for training, validating and testing of single-step model.

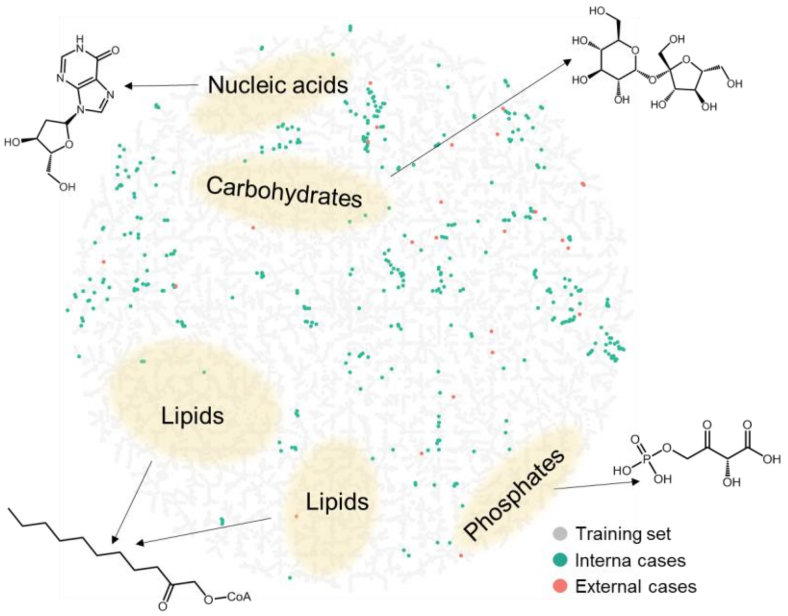

Supplementary Figure 4. Chemical space of training set, internal cases and external cases for multi-step planning. The clustering and visualization of chemical space was realized by TMAP[13]. The biosynthesis of long chain fatty acid (lipids), carbohydrates, nucleic acids, phosphates is not considered in current work. An interactive web page (http://biopathnavi.qmclab.com/tmap.html) is provided to see the nearest-neighbor of cases in the training set.

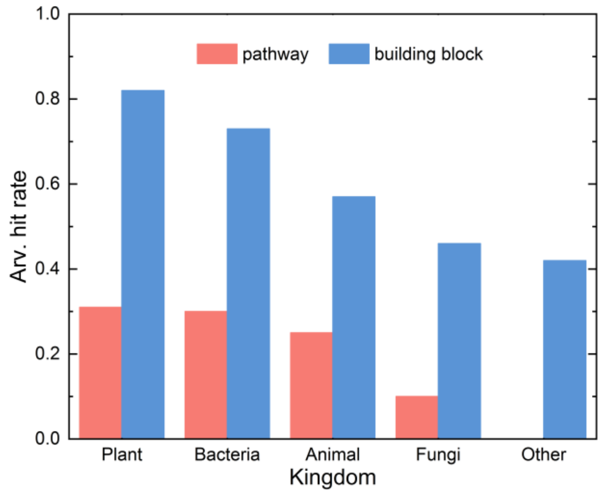

Supplementary Figure 5. The BioNavi-NP's performance within each kingdom. Source data are provided as a Source Data file.

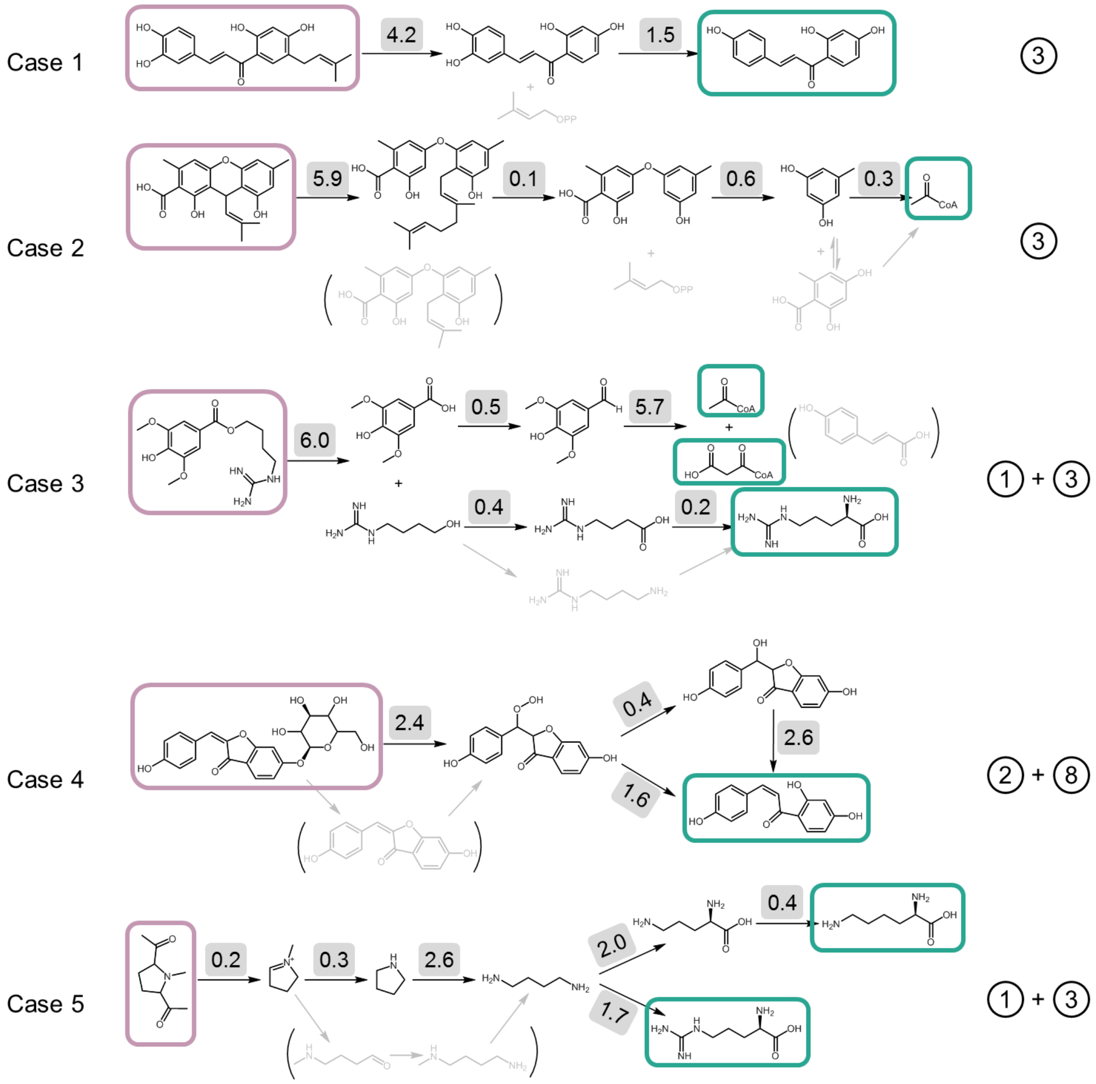

Supplementary Figure 6. Five examples of the 25 cases in the external test set. Target molecules are in purple box and building blocks in green box. The output pathways (max number of pathways was set to 10) were integrated manually and the missing or unreasonable intermediates were colored gray. The cost of each step is given in the gray block and the rank is shown at the end of the pathway. The complete predicted pathways could be found by http://biopathnavi.qmclab.com/case.html.

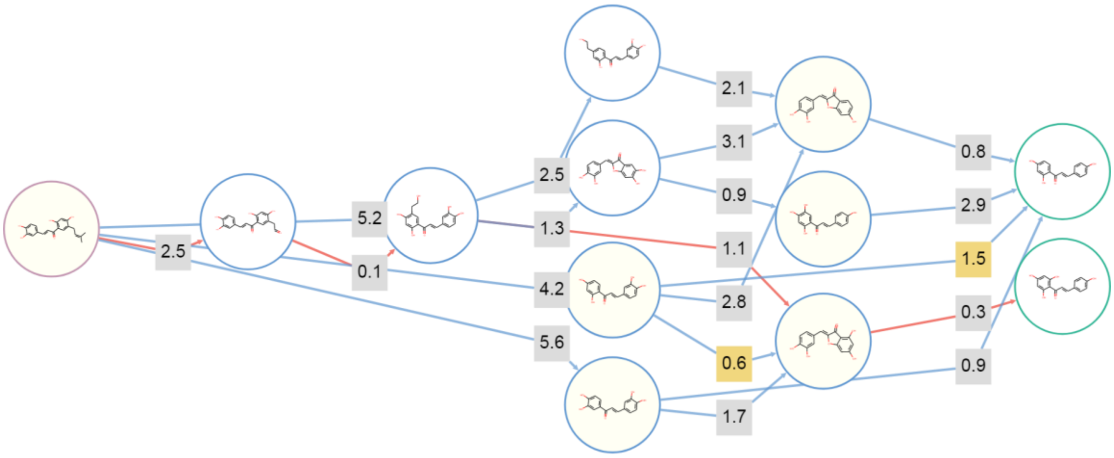

Supplementary Figure 7. The complete output of case 1 by BioNavi-NP webserver. The max number of pathways was set to 10. Target molecule is highlighted in purple and building blocks are in green. A Web version can be found by http://biopathnavi.qmclab.com/job.html?JobId=CASE1

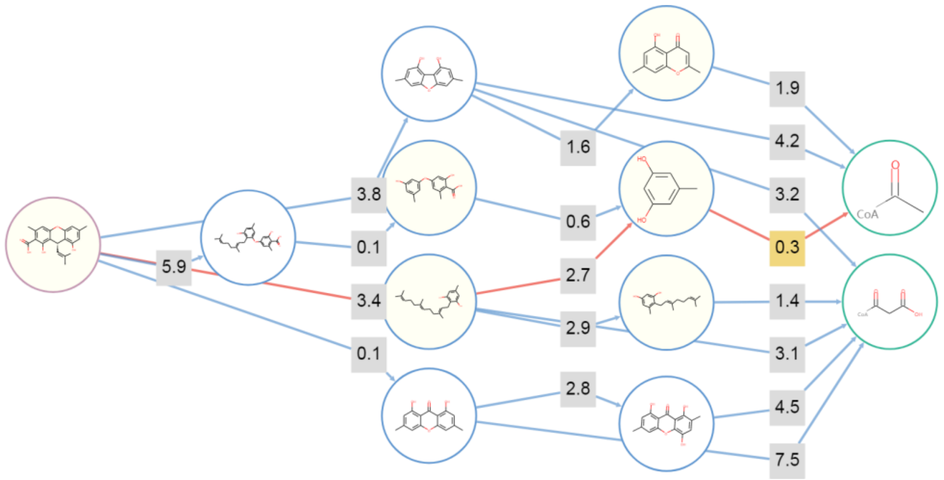

Supplementary Figure 8. The complete output of case 2 by BioNavi-NP webserver. The max number of pathways was set to 10. Target molecules are highlighted in purple and building blocks in green. A Web version can be found by http://biopathnavi.qmclab.com/job.html?JobId=CASE2

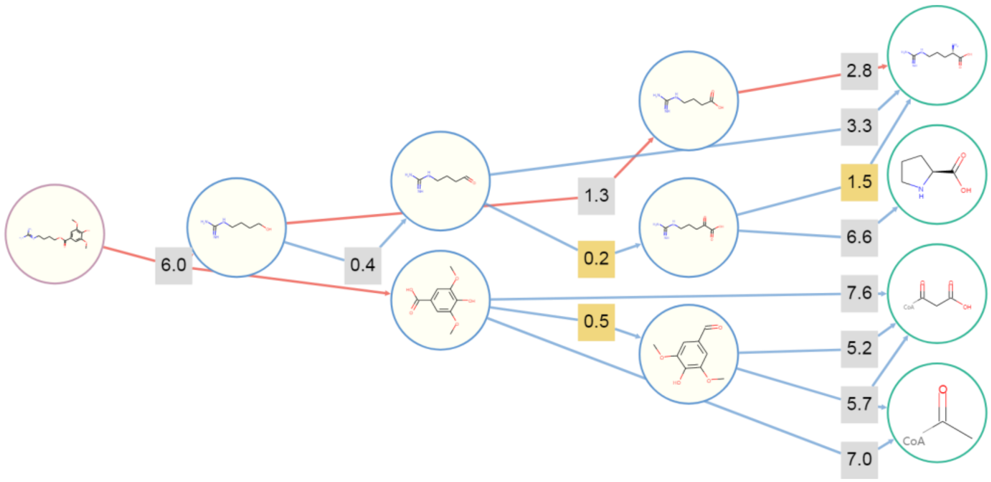

Supplementary Figure 9. The complete output of case 3 by BioNavi-NP webserver. The max number of pathways was set to 10. Target molecules are highlighted in purple and building blocks in green. A Web version can be found by http://biopathnavi.qmclab.com/job.html?JobId=CASE3

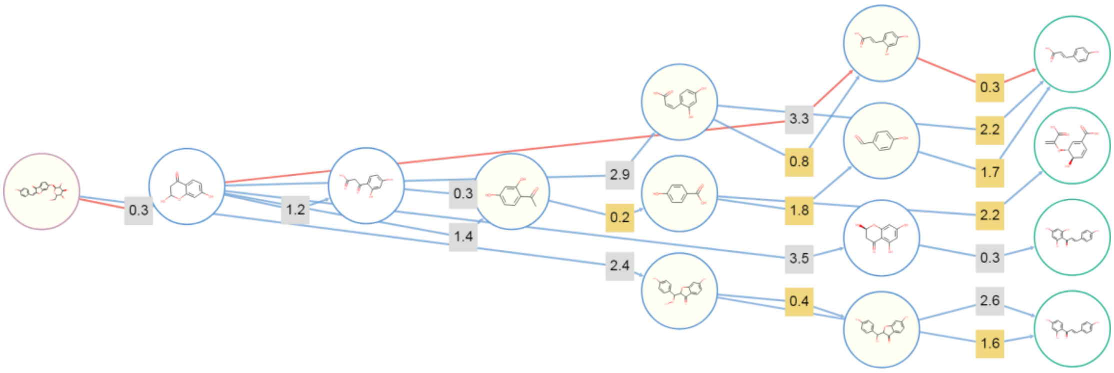

Supplementary Figure 10. The complete output of case 4 by BioNavi-NP webserver. The max number of pathways was set to 10. Target molecules are highlighted in purple and building blocks in green. A Web version can be found by http://biopathnavi.qmclab.com/job.html?JobId=CASE4

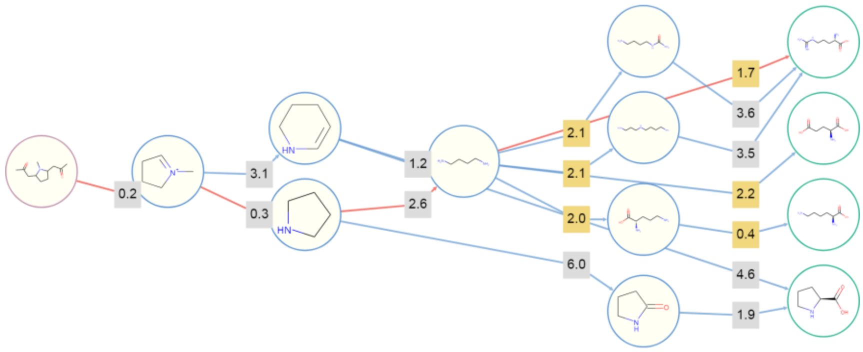

Supplementary Figure 11. The complete output of case 5 by BioNavi-NP webserver. The max number of pathways was set to 10. Target molecules are highlighted in purple and building blocks in green. A Web version can be found by http://biopathnavi.qmclab.com/job.html?JobId=CASE5

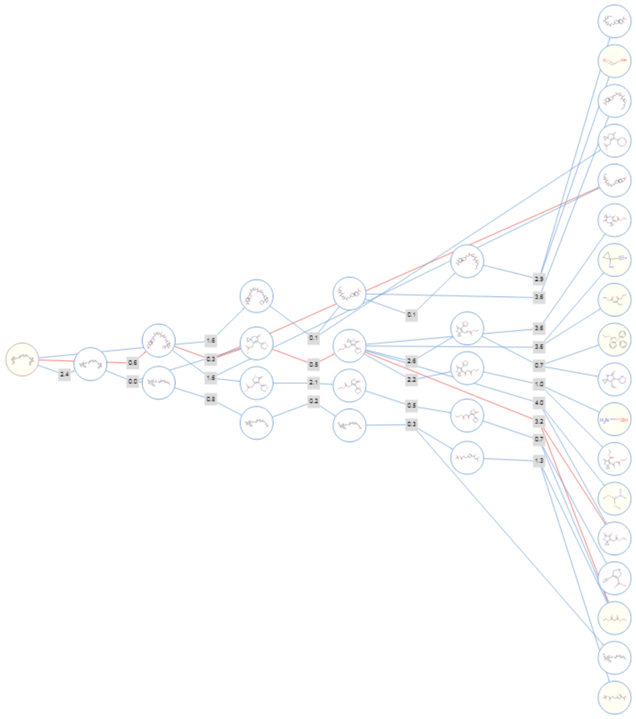

Supplementary Figure 12. The complete output of case 6 by BioNavi-NP webserver. The max number of pathways was set to 10. Target molecule is highlighted in purple and building blocks are in green. A Web version can be found by http://biopathnavi.qmclab.com/job.html?JobId=CASE6. No pathway was found between the target structure and given building blocks, and the incomplete pathways were output.

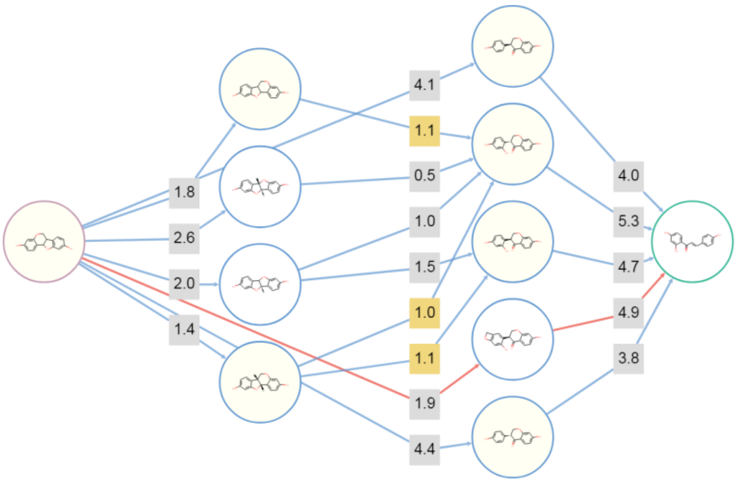

Supplementary Figure 13. The complete output of case 7 by BioNavi-NP webserver. The max number of pathways was set to 10. Target molecules are highlighted in purple and building blocks in green. A Web version can be found by http://biopathnavi.qmclab.com/job.html?JobId=CASE7

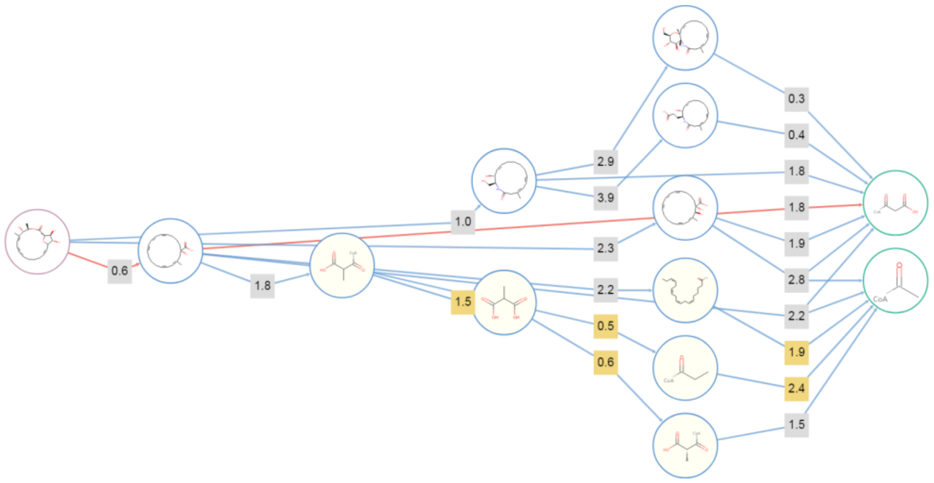

Supplementary Figure 14. The complete output of case 8 by BioNavi-NP webserver. The max number of pathways was set to 10. Target molecules are highlighted in purple and building blocks in green. A Web version can be found by http://biopathnavi.qmclab.com/job.html?JobId=CASE8

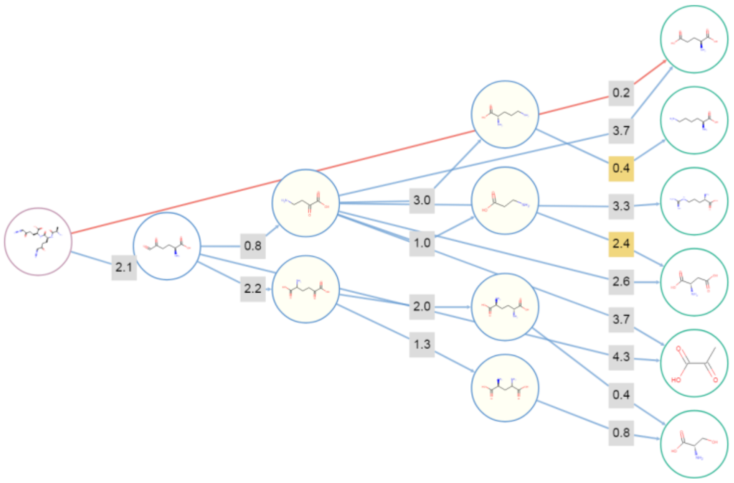

Supplementary Figure 15. The complete output of case 9 by BioNavi-NP webserver. The max number of pathways was set to 10. Target molecules are highlighted in purple and building blocks in green. A Web version can be found by http://biopathnavi.qmclab.com/job.html?JobId=CASE9

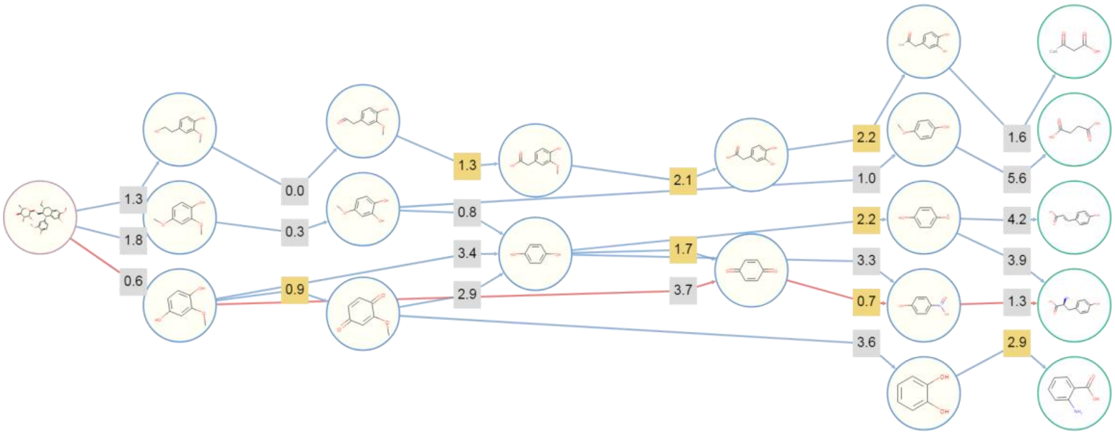

Supplementary Figure 16. The complete output of case 10 by BioNavi-NP webserver. The max number of pathways was set to 10. Target molecules are highlighted in purple and building blocks in green. A Web version can be found by http://biopathnavi.qmclab.com/job.html?JobId=CASE10

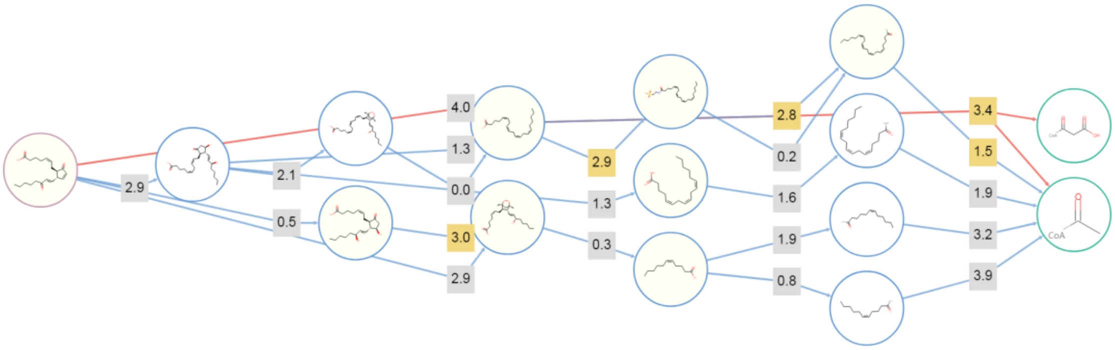

Supplementary Figure 17. The complete output of case 11 by BioNavi-NP webserver. The max number of pathways was set to 10. Target molecules are highlighted in purple and building blocks in green. A Web version can be found by http://biopathnavi.qmclab.com/job.html?JobId=CASE11

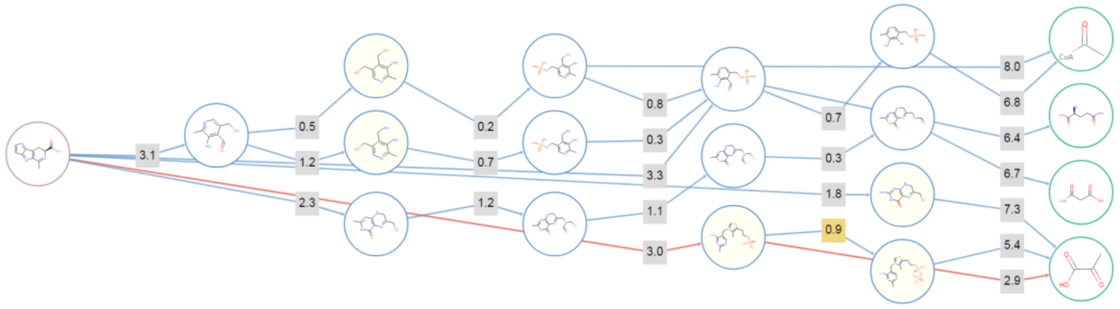

Supplementary Figure 18. The complete output of case 12 by BioNavi-NP webserver. The max number of pathways was set to 10. Target molecules are highlighted in purple and building blocks in green. A Web version can be found by

http://biopathnavi.qmclab.com/job.html?JobId=CASE12

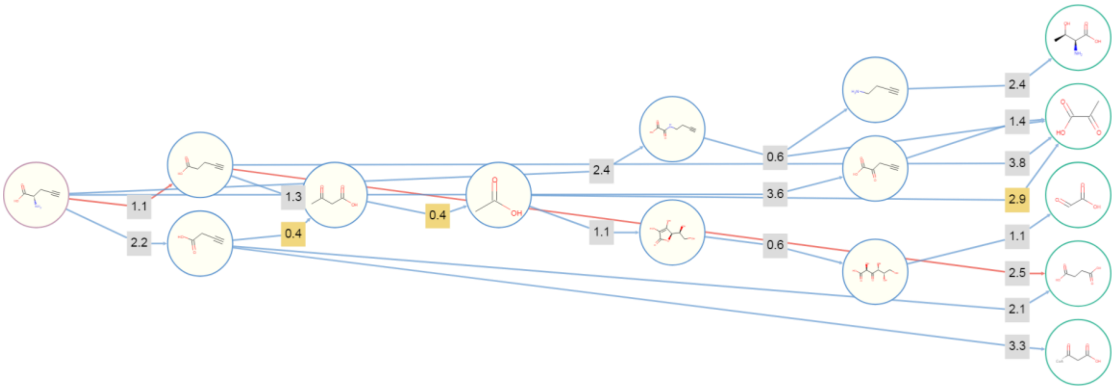

Supplementary Figure 19. The complete output of case 13 by BioNavi-NP webserver. The max number of pathways was set to 10. Target molecules are highlighted in purple and building blocks in green. A Web version can be found by http://biopathnavi.qmclab.com/job.html?JobId=CASE13

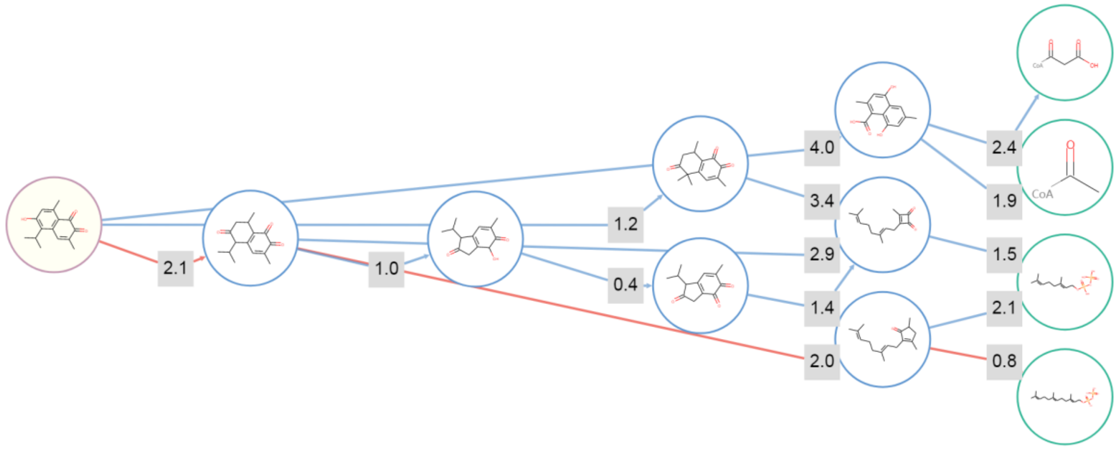

Supplementary Figure 20. The complete output of case 14 by BioNavi-NP webserver. The max number of pathways was set to 10. Target molecules are highlighted in purple and building blocks in green. A Web version can be found by http://biopathnavi.qmclab.com/job.html?JobId=CASE14

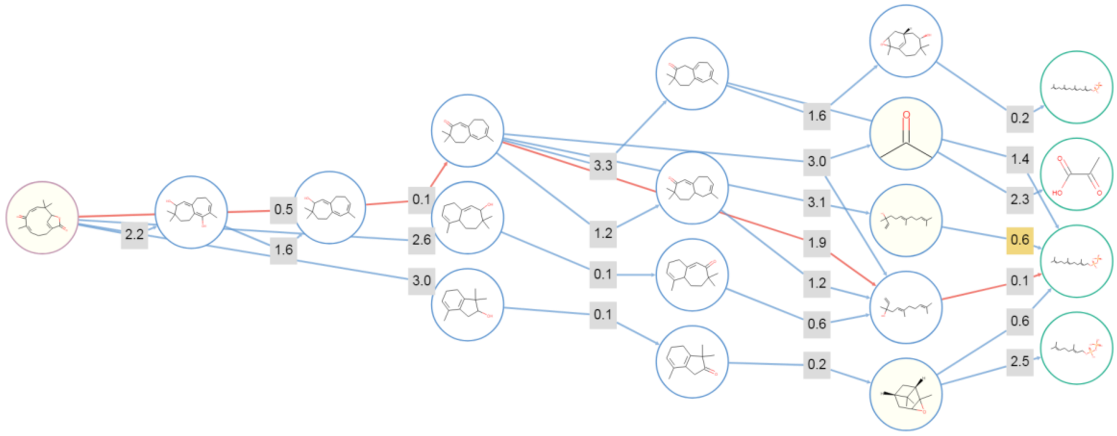

Supplementary Figure 21. The complete output of case 15 by BioNavi-NP webserver. The max number of pathways was set to 10. Target molecules are highlighted in purple and building blocks in green. A Web version can be found by http://biopathnavi.qmclab.com/job.html?JobId=CASE15

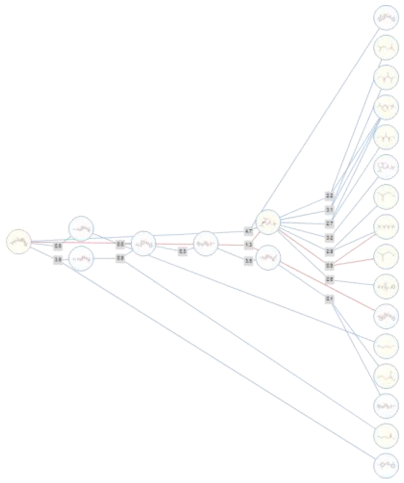

Supplementary Figure 22. The complete output of case 16 by BioNavi-NP webserver. The max number of pathways was set to 10. Target molecules are highlighted in purple and building blocks in green. A Web version can be found by http://biopathnavi.qmclab.com/job.html?JobId=CASE16. No pathway was found between the target structure and given building blocks, and the incomplete pathways were output.

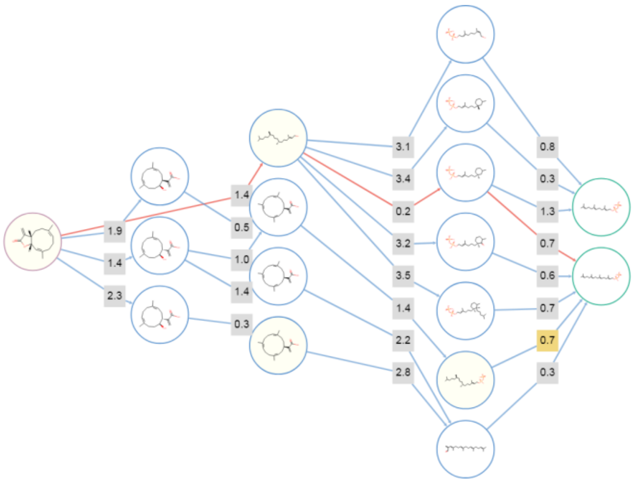

Supplementary Figure 23. The complete output of case 17 by BioNavi-NP webserver. The max number of pathways was set to 10. Target molecules are highlighted in purple and building blocks in green. A Web version can be found by http://biopathnavi.qmclab.com/job.html?JobId=CASE17

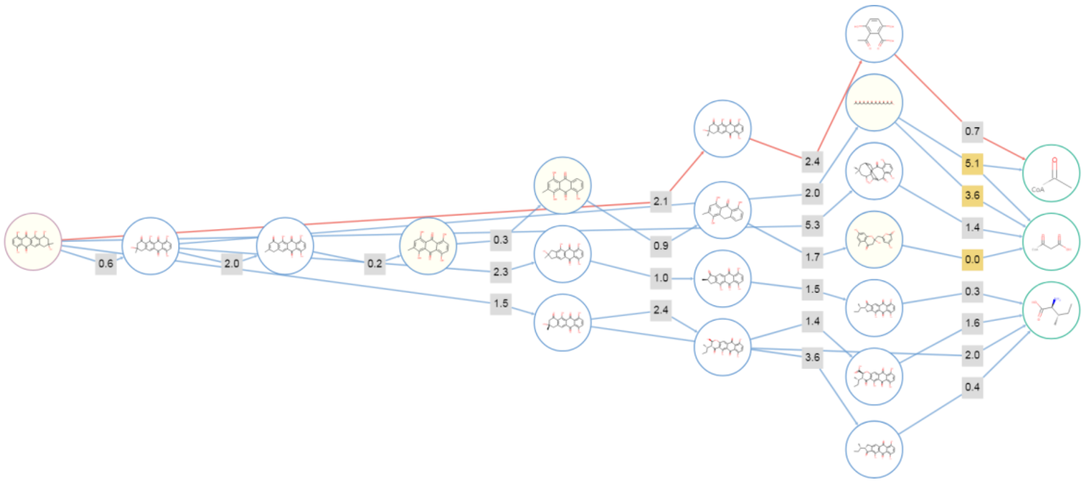

Supplementary Figure 24. The complete output of case 18 by BioNavi-NP webserver. The max number of pathways was set to 10. Target molecules are highlighted in purple and building blocks in green. A Web version can be found by

http://biopathnavi.qmclab.com/job.html?JobId=CASE18

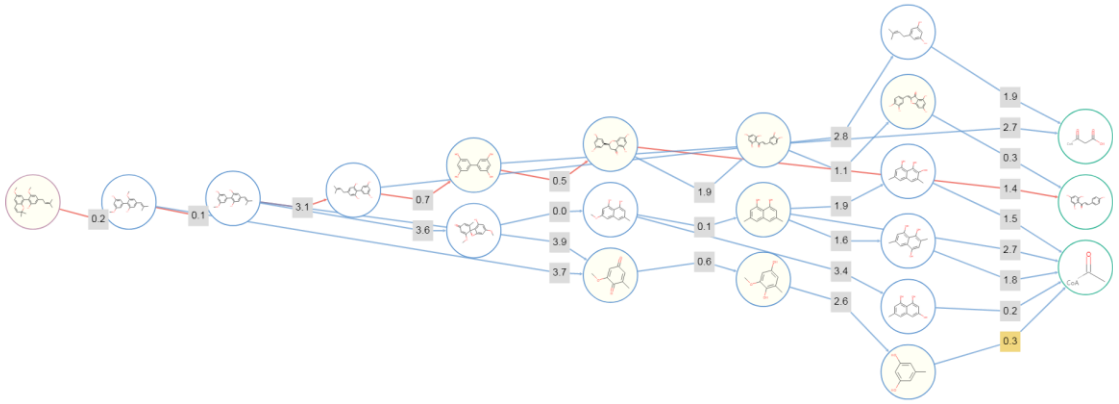

Supplementary Figure 25. The complete output of case 19 by BioNavi-NP webserver. The max number of pathways was set to 10. Target molecules are highlighted in purple and building blocks in green. A Web version can be found by

http://biopathnavi.qmclab.com/job.html?JobId=CASE19

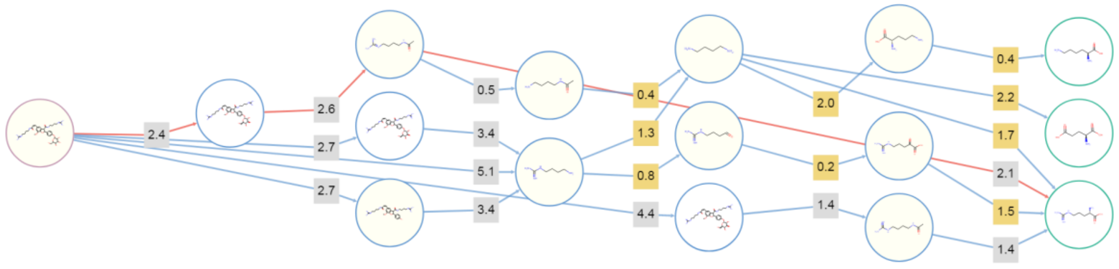

Supplementary Figure 26. The complete output of case 20 by BioNavi-NP webserver. The max number of pathways was set to 10. Target molecules are highlighted in purple and building blocks in green. A Web version can be found by http://biopathnavi.qmclab.com/job.html?JobId=CASE20

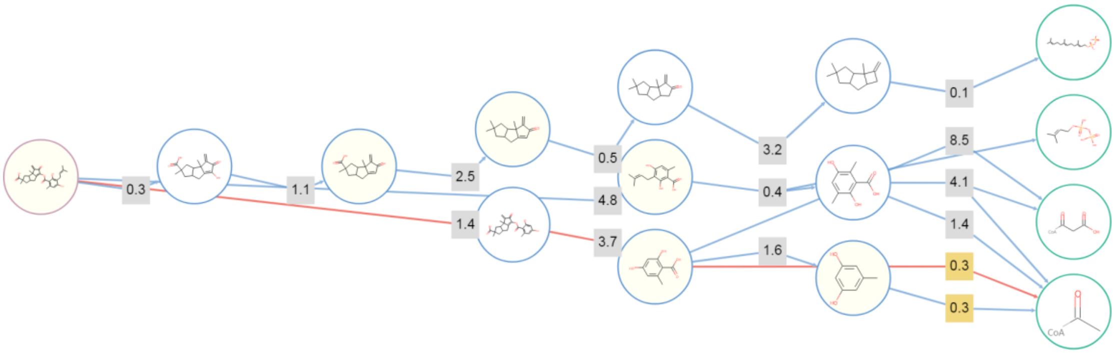

Supplementary Figure 27. The complete output of case 21 by BioNavi-NP webserver. The max number of pathways was set to 10. Target molecules are highlighted in purple and building blocks in green. A Web version can be found by

http://biopathnavi.qmclab.com/job.html?JobId=CASE21

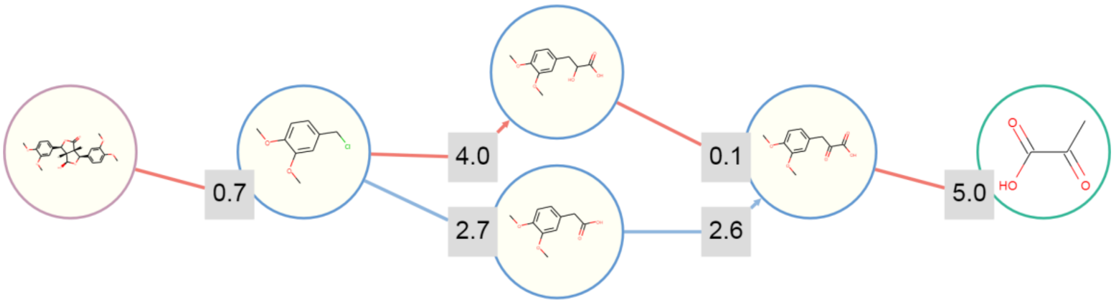

Supplementary Figure 28. The complete output of case 22 by BioNavi-NP webserver. The max number of pathways was set to 10. Target molecules are highlighted in purple and building blocks in green. A Web version can be found by http://biopathnavi.qmclab.com/job.html?JobId=CASE22

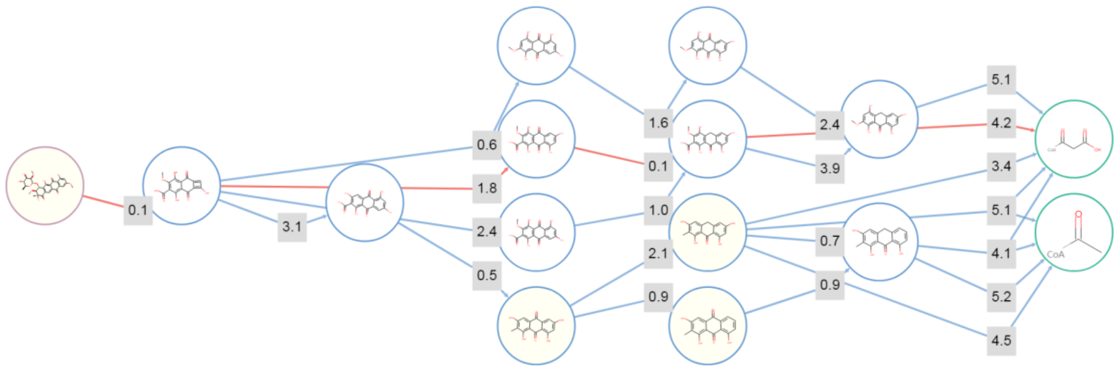

Supplementary Figure 29. The complete output of case 23 by BioNavi-NP webserver. The max number of pathways was set to 10. Target molecules are highlighted in purple and building blocks in green. A Web version can be found by http://biopathnavi.qmclab.com/job.html?JobId=CASE23

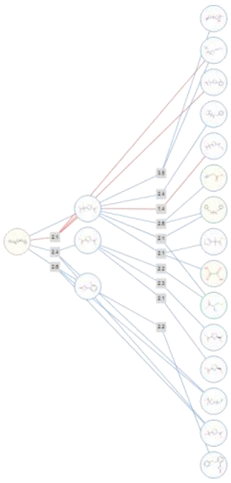

Supplementary Figure 30. The complete output of case 24 by BioNavi-NP webserver. The max number of pathways was set to 10. Target molecules are highlighted in purple and building blocks in green. A Web version can be found by http://biopathnavi.qmclab.com/job.html?JobId=CASE24. No pathway was found between the target structure and given building blocks, and the incomplete pathways were output.

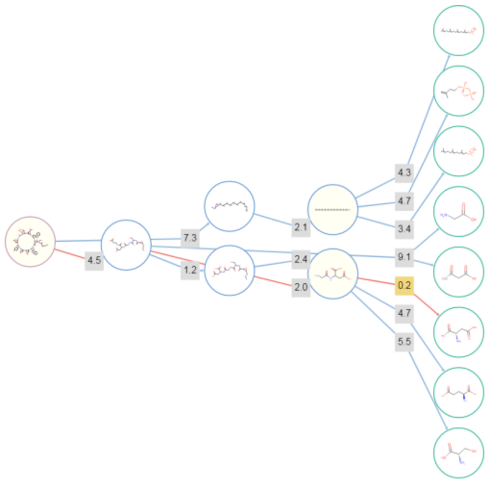

Supplementary Figure 31. The complete output of case 25 by BioNavi-NP webserver. The max number of pathways was set to 10. Target molecules are highlighted in purple and building blocks in green. A Web version can be found by http://biopathnavi.qmclab.com/job.html?JobId=CASE25.

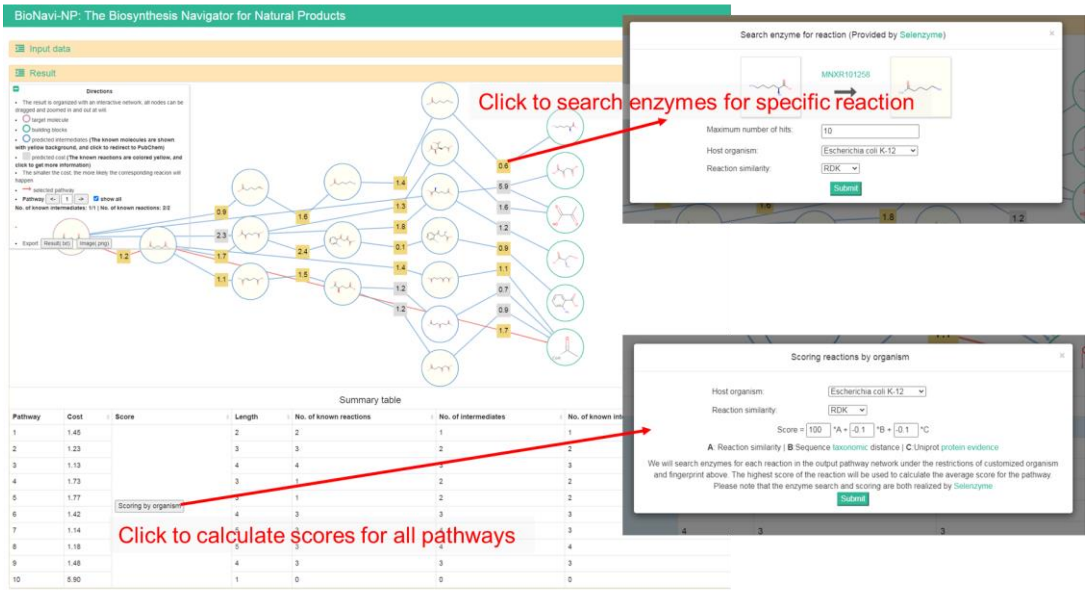

Supplementary Figure 32. The result panel of BioNavi-NP. It consists of the interactive pathway network and the summary table, where the pathways can be re-ranked by serval items. Selenzym[14] was accessed to search enzymes and score for specific reactions.

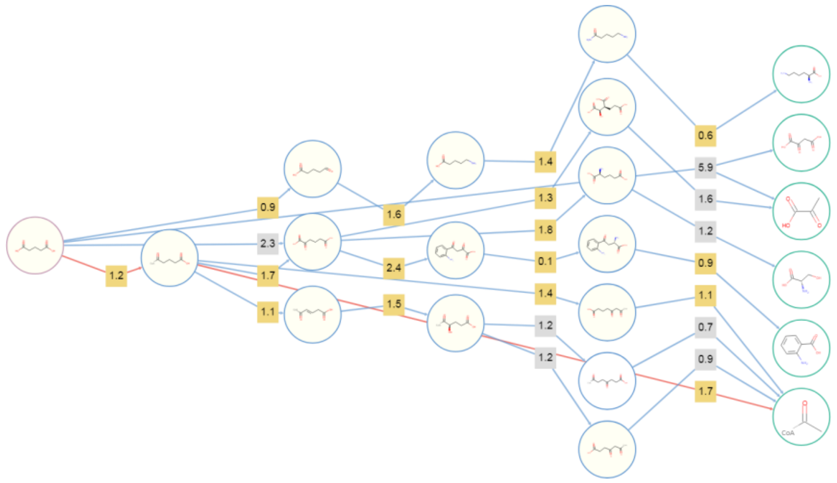

Supplementary Figure 33. The complete output of glutarate by BioNavi-NP webserver. The max number of pathways was set to 10. Target molecules are highlighted in purple and building blocks in green. A Web version can be found by http://biopathnavi.qmclab.com/job.html?JobId=CASE26.

Supplementary references

1. Vaswani A*, et al.* Attention is all you need. In: *Proceedings of the 31st International Conference on Neural Information Processing Systems*. Curran Associates Inc. (2017).
2. Chen B, Li C, Dai H, Song L. Retro*: learning retrosynthetic planning with neural guided A* search. In: *International Conference on Machine Learning*. PMLR (2020).
3. Zheng S, Rao J, Zhang Z, Xu J, Yang Y. Predicting retrosynthetic reactions using self-corrected transformer neural networks. *J Chem Inf Model* **60**, 47-55 (2020).
4. Tillmann C, Ney H. Word reordering and a dynamic programming beam search algorithm for statistical machine translation. *Comput Linguist* **29**, 97-133 (2003).
5. Kuwahara H, Alazmi M, Cui X, Gao X. MRE: a web tool to suggest foreign enzymes for the biosynthesis pathway design with competing endogenous reactions in mind. *Nucleic Acids Res* **44**, W217-W225 (2016).
6. Latendresse M, Krummenacker M, Karp PD. Optimal metabolic route search based on atom mappings. *Bioinformatics* **30**, 2043-2050 (2014).
7. Moriya Y*, et al.* PathPred: an enzyme-catalyzed metabolic pathway prediction server. *Nucleic Acids Res* **38**, W138-W143 (2010).
8. Kumar A, Wang L, Ng CY, Maranas CD. Pathway design using de novo steps through uncharted biochemical spaces. *Nat Commun* **9**, 184 (2018).
9. Wicker J*, et al.* enviPath – The environmental contaminant biotransformation pathway resource. *Nucleic Acids Res* **44**, D502-D508 (2016).
10. Hatzimanikatis V, Li C, Ionita JA, Henry CS, Jankowski MD, Broadbelt LJ. Exploring the diversity of complex metabolic networks. *Bioinformatics* **21**, 1603-1609 (2005).
11. MohammadiPeyhani H, Hafner J, Sveshnikova A, Viterbo V, Hatzimanikatis V. Expanding biochemical knowledge and illuminating metabolic dark matter with ATLASx. *Nat Commun* **13**, 1560 (2022).
12. Koch M, Duigou T, Faulon JL. Reinforcement learning for bioretrosynthesis. *ACS Synth Biol* **9**, 157-168 (2020).
13. Probst D, Reymond J-L. Visualization of very large high-dimensional data sets as minimum spanning trees. *J Cheminf* **12**, 12 (2020).
14. Carbonell P*, et al.* Selenzyme: enzyme selection tool for pathway design. *Bioinformatics* **34**, 2153-2154 (2018).
