# Supplementary material for: Deep learning driven biosynthetic pathways navigation for natural products with BioNavi-NP
Source: Nat Commun. 2022 Jun 10;13:3342. doi: 10.1038/s41467-022-30970-9 (PMC9187661; doi:10.1038/s41467-022-30970-9)
Supplement: Supplementary file 3 — Reporting Summary [file 41467_2022_30970_MOESM3_ESM.pdf]

nature research

Corresponding author(s): Ruibo Wu

Last updated by author(s): May 24, 2022

Reporting Summary

Nature Research wishes to improve the reproducibility of the work that we publish. This form provides structure for consistency and transparency in reporting. For further information on Nature Research policies, see our Editorial Policies and the Editorial Policy Checklist.

Statistics

For all statistical analyses, confirm that the following items are present in the figure legend, table legend, main text, or Methods section.

n/a	Confirmed	
☒	☐	The exact sample size (*n*) for each experimental group/condition, given as a discrete number and unit of measurement
☒	☐	A statement on whether measurements were taken from distinct samples or whether the same sample was measured repeatedly
☒	☐	The statistical test(s) used AND whether they are one- or two-sided<br>*Only common tests should be described solely by name; describe more complex techniques in the Methods section.*
☒	☐	A description of all covariates tested
☒	☐	A description of any assumptions or corrections, such as tests of normality and adjustment for multiple comparisons
☒	☐	A full description of the statistical parameters including central tendency (e.g. means) or other basic estimates (e.g. regression coefficient) AND variation (e.g. standard deviation) or associated estimates of uncertainty (e.g. confidence intervals)
☒	☐	For null hypothesis testing, the test statistic (e.g. *F*, *t*, *r*) with confidence intervals, effect sizes, degrees of freedom and *P* value noted<br>*Give P values as exact values whenever suitable.*
☒	☐	For Bayesian analysis, information on the choice of priors and Markov chain Monte Carlo settings
☒	☐	For hierarchical and complex designs, identification of the appropriate level for tests and full reporting of outcomes
☒	☐	Estimates of effect sizes (e.g. Cohen's *d*, Pearson's *r*), indicating how they were calculated

Our web collection on statistics for biologists contains articles on many of the points above.

Software and code

Policy information about availability of computer code

Data collection	RDKit package (release 2020.03.6) for molecule structure preparation. Pytorch (version 1.7.0) and OpenNMT (version2.2.0) for Transformer neural networks construction.
Data analysis	TMAP (version 1.0.4) for molecular clustering and visualization.

For manuscripts utilizing custom algorithms or software that are central to the research but not yet described in published literature, software must be made available to editors and reviewers. We strongly encourage code deposition in a community repository (e.g. GitHub). See the Nature Research guidelines for submitting code & software for further information.

Data

Policy information about availability of data

All manuscripts must include a data availability statement. This statement should provide the following information, where applicable:

- Accession codes, unique identifiers, or web links for publicly available datasets
- A list of figures that have associated raw data
- A description of any restrictions on data availability

The raw data is collected from KEGG (https://www.kegg.jp/), MeatCyc (https://metacyc.org/) and MetaNetX (https://www.metanetx.org/). The processed data that used to train and test the model is available at http://biopathnavi.qmclab.com/data.html. The raw output of external cases are available at http://biopathnavi.qmclab.com/case.html.

Field-specific reporting

Please select the one below that is the best fit for your research. If you are not sure, read the appropriate sections before making your selection.

☒ Life sciences ☐ Behavioural & social sciences ☐ Ecological, evolutionary & environmental sciences

For a reference copy of the document with all sections, see nature.com/documents/nr-reporting-summary-flat.pdf

Life sciences study design

All studies must disclose on these points even when the disclosure is negative.

Sample size	All reactions from MetaNetX, KEGG and MetaCyc were collected, and those with unavailable SMILES were removed, resulting 33710 biochemical reactions. 62370 organic reactions containing natural products-like components from USPTO were selected. The organic reactions can help the model learn the general patterns when less biochemical data are available.
Data exclusions	The biochemical reactions with unavailable SMILES were excluded since the model use SMILES as molecule representation.
Replication	Computational experiments may be reproduced using the code and data; see Code and Data Availability sections
Randomization	The dataset was split randomly.
Blinding	The test was performed on computational models, in which blinding was not relevant.

Reporting for specific materials, systems and methods

We require information from authors about some types of materials, experimental systems and methods used in many studies. Here, indicate whether each material, system or method listed is relevant to your study. If you are not sure if a list item applies to your research, read the appropriate section before selecting a response.

Materials & experimental systems

n/a	Involved in the study
☒	☐ Antibodies
☒	☐ Eukaryotic cell lines
☒	☐ Palaeontology and archaeology
☒	☐ Animals and other organisms
☒	☐ Human research participants
☒	☐ Clinical data
☒	☐ Dual use research of concern

Methods

n/a	Involved in the study
☒	☐ ChIP-seq
☒	☐ Flow cytometry
☒	☐ MRI-based neuroimaging
